# Effect of Organic Acid Selection on the Physicochemical Properties, Bioadhesion, and Stability of Chitosan Hydrogels

**DOI:** 10.3390/gels11100778

**Published:** 2025-09-28

**Authors:** Muhammet Davut Arpa, Ümit Can Erim, Ebrar Elif Kesmen Salik, Sevde Nur Biltekin Kaleli, Ismail Erol

**Affiliations:** 1 Department of Pharmaceutical Technology, School of Pharmacy, Istanbul Medipol University, 34815 Istanbul, Türkiye; 2 Department of Analytical Chemistry, School of Pharmacy, Istanbul Medipol University, 34815 Istanbul, Türkiye; 3 Department of Pharmaceutical Technology, Faculty of Pharmacy, University of Health Sciences, 34668 Istanbul, Türkiye; 4 Department of Microbiology, School of Pharmacy, Istanbul Medipol University, 34815 Istanbul, Türkiye; 5 Department of Analytical Chemistry, School of Pharmacy, Bahçeşehir University, 34353 Istanbul, Türkiye

**Keywords:** chitosan, chitosan salt, hydrogel, bioadhesion, organic acid, stability

## Abstract

Chitosan is a promising biopolymer for drug delivery due to its biocompatibility, biodegradability, and low toxicity. However, its limited dispersibility in water restricts applications, which can be improved through organic acid salts. This study examined how acetic, lactic, glutamic, and citric acids influence the physicochemical, rheological, swelling, bioadhesive, stability, and cytotoxicity properties of chitosan hydrogels. Gels were prepared using varying chitosan-to-acid molar ratios (1:1; 1:1.2 for citrate) and characterized by NMR, FTIR, TGA, and XRD. Despite identical chitosan concentrations (2%, 3%, 3.5%), gels displayed distinct viscosity, swelling, and adhesion profiles depending on the acid. Lactate gels exhibited the most favorable overall performance, combining high viscosity (1555–6665 mPa·s), structural stability, and strong bioadhesion. Citrate gels showed the lowest viscosity (825–3550 mPa·s), cell viability, and stability but the highest bioadhesiveness, likely due to multivalent ionic interactions. Short-term stability tests revealed that low pH accelerated chitosan degradation, leading to viscosity loss up to ~90–95% within 30 days, particularly in citrate hydrogels. Cytotoxicity tests confirmed high biocompatibility, with all formulations maintaining cell viability above 80%. Overall, the findings highlight that organic acid selection is a critical determinant of chitosan gel behavior, offering guidance for tailoring safe, stable, and bioadhesive drug delivery systems.

## 1. Introduction

Chitosan, which is a copolymer consisting of *β*-1,4-linked D-glucosamine and *N*-acetyl-D-glucosamine units obtained by partial deacetylation of chitin in an alkaline medium, is the only naturally occurring cationic polysaccharide [1,2,3]. With its unique properties of biocompatibility, biodegradability, low toxicity, and antibacterial activity, chitosan, which is predominantly extracted from crustacea such as crab, prawn, and shrimp, has been commonly used in pharmaceutical, biomedical, food, and cosmetic fields [4,5,6]. In addition, positively charged chitosan exhibits good bioadhesive properties by interacting electrostatically with negatively charged mucin and thus enhances the absorption of drugs by providing prolonged contact time [7]. In recent years, many chitosan derivatives chemically modified, such as trimethyl chitosan [8], carboxymethyl chitosan [9], thiolated chitosan [10,11], acrylated chitosan [12], and quaternary ammonium chitosan [13] have been synthesized to further improve these unique properties of chitosan. However, the main obstacle to providing all these advantages of chitosan is its limited dispersibility in neutral and alkaline aqueous environments, which prevents chain repulsion and leads to aggregation. While intermolecular hydrogen bonding and high molecular weight may further reduce mobility, the primary barrier is its pH-dependent charge density [14,15,16,17]. In general, to achieve the aqueous dispersibility of chitosan, two ways have been focused on: degrading chitosan to oligochitosan (2–10 monomer units) or forming chitosan salts [18]. For the latter way, the chitosan salt, or salified chitosan, is obtained by using dilute organic or inorganic acids at different molar concentrations [19,20]. The goal is to ensure the dispersibility with a sufficient amount of acid to protonate the amine groups in the glucosamine units of chitosan. One of the most widely used organic acids is acetic acid for this purpose [21,22,23,24]. Aspartic acid, glutamic acid, lactic acid, ascorbic acid, and hydrochloric acid with different chemical structures and ionic strength are also commonly preferred [16,25,26,27,28,29,30]. These acids are used as aqueous solutions in different molar ratios such as 0.1, 0.5, 0.75, 1, 1.5, 2, 3, and 5 versus one mol glucosamine unit of chitosan [16,20,31,32,33,34,35]. The molecular weight and the degree of deacetylation of chitosan affect its dispersibility. However, the type, ionic strength, pK_a_, and concentration of acids directly affect chitosan’s dispersibility and all the physicochemical properties of the obtained chitosan-based dispersion/gel, especially the rheological properties and the stability [26,35,36,37].

Chitosan needs an acidic environment (pH < 6.5) to disperse; however, an excessively low pH can significantly reduce its molecular weight over time [35]. That is, when chitosan is exposed to concentrated acids or a large amount of acid is used, a large amount of oligochitosan distribution occurs [38,39]. For this reason, it is very difficult to keep chitosan dispersions stable at room temperature for a long time [35]. Up to now, while most of the studies have focused on the dispersibility and dispersion behavior of dilute chitosan aqueous dispersions using rheology and light scattering methods [16,23,36,40,41], studies on the effects of acid type in chitosan dispersion/gel on physicochemical properties and stability are still insufficient in the pharmaceutical area.

In this work, it is hypothesized that the use of different organic acids (acetic acid, citric acid, glutamic acid, and lactic acid) will cause significant changes in the physicochemical character of chitosan hydrogels, and variations in chitosan concentration and acid type may affect the physical stability of the gel. As a result, it is expected that this situation will cause significant changes in the effectiveness of chitosan-based drug delivery systems. To this end, the rheological character, pH, cytotoxicity, bioadhesion, and swelling properties of chitosan gels prepared with four different organic acids were investigated. Furthermore, the short-term physical stability of the formulations was assessed by monitoring viscosity changes over a 30-day period, focusing on the influence of both the type of organic acid and chitosan concentration, which are critical formulation variables in gel systems.

## 2. Results and Discussion

### 2.1. Preparation of Chitosan Hydrogels

**Figure 1:**
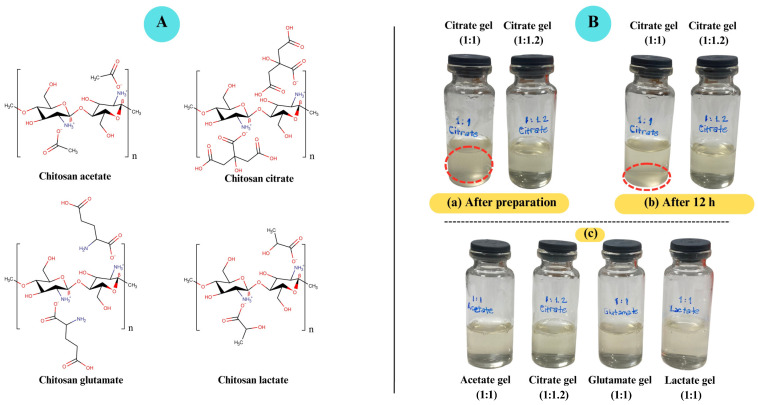
(**A**). Chitosan salts (chitosan acetate, chitosan citrate, chitosan glutamate, chitosan lactate). (**B**). Visual appearance of chitosan salt-based gels. (**a**) Chitosan–citric acid gels at 1:1 and 1:1.2 molar ratios immediately after preparation. (**b**) Chitosan citrate gels after 12 h. (**c**) Chitosan salt-based gels immediately after preparation. Chitosan citrate gels prepared at a 1:1 molar ratio appeared turbid and exhibited sedimentation after 12 h, whereas citrate gels at 1:1.2 remained fully dispersed and clear. In contrast, gels prepared with acetic, glutamic, and lactic acids at 1:1 ratios were clear and stable.

The structure of chitosan–acid salts is illustrated in Figure 1. Chitosan salt gels (Figure 1B) were prepared using organic acids such as acetic acid, ascorbic acid, citric acid, glutamic acid, and lactic acid, which protonate the amino groups of chitosan to facilitate dispersibility. The composition of the hydrogels is demonstrated in Table 1. Chitosan dispersed rapidly with ascorbic acid, producing transparent gels. However, these gels lost their viscous character within a few days, transitioning to a sol system, and their color changed from yellowish to brown. This degradation was attributed to hydroperoxyl radicals generated by the reaction of ascorbic acid with atmospheric oxygen [42]. Consequently, chitosan ascorbate gels were excluded from the study due to poor physical stability.

Previous studies typically used a 1:1 molar ratio of organic acids with amino acid structures for chitosan dispersibility [26,28,43]. Acetic acid, however, was used in varying molar ratios [21,22,23,24]. Visual assessments confirmed that 1 mol of acid per 1 mol of glucosamine unit was sufficient to disperse the chitosan (low-molecular-weight (L-MW) and 90% deacetylation degree (DD)) used in this study, except for citric acid.

Chitosan’s MW and DD are of great importance for its dispersibility. Gupta (2007) [44] demonstrated that L-MW chitosan dispersed within 2 h, while high-molecular-weight (H-MW) chitosan dispersed within 24 h using 2% acetic acid. Furthermore, chitosan with high DD (75%) dispersed more readily than chitosan with low DD (48%). Additionally, Furuike (2017) [35] and Iftime (2020) [43] used acetic acid at a molar ratio of 1:1 to disperse chitosan with a DD of 77–85% and a medium MW (M-MW) and/or L-MW in their studies. These findings align with our results, reaffirming that MW and DD are critical for controlling chitosan–solvent interactions.

To prepare the chitosan gels, the acid was first dissolved in water, followed by the addition of chitosan to the acid solution, and mixed to obtain clear gels. Since chitosan does not dissolve in pure water without protonation, this step ensures complete solubilization and homogeneous gel formation [17,28,45]. While gels prepared with lactic acid and acetic acid were easily obtained, the preparation of chitosan glutamate gels required adding glutamic acid and chitosan to water simultaneously. Glutamic acid, being a weak acid, has limited solubility in water at room temperature [46,47]. However, when mixed together, chitosan and glutamic acid readily form chitosan glutamate salt. The distinction between the preparation of chitosan gels using acetic acid, citric acid, and lactic acid versus glutamic acid lies in the physicochemical behavior of these acids in aqueous environments. Glutamic acid has a much lower water solubility compared to the others [48], and it acts as a zwitterionic compound under physiological pH, limiting its proton-donating capacity. In contrast, citric acid has a higher ionic strength and buffering capacity, which required a slightly higher molar ratio (1:1.2, chitosan:acid) to ensure full protonation of the glucosamine units. This increased protonation improves chitosan solubilization and contributes to the formation of a homogeneous gel. In the case of glutamic acid, although only partially soluble, the available H_3_O^+^ ions are sufficient to protonate chitosan, while the remaining undissolved glutamic acid particles contribute to ion–ion interactions with protonated amines, enabling stable gel formation.

Citric acid, a common metabolite in plants and animals, is a weak organic acid with a tricarboxylic structure and good water solubility [49,50]. However, when citric acid was used at a 1:1 molar ratio with chitosan, a clear dispersion was not achieved. A cloudy dispersion formed, while a portion of the chitosan failed to disperse and was observed as precipitated material. By incrementally adding citric acid to the dispersion, it was determined that a molar ratio of 1.2 was required for a clear dispersion of chitosan (Figure 1B). Additionally, while the gels prepared with other acids were successfully obtained at room temperature, the chitosan citrate gels required preparation at 50 °C. This aligns with previous studies, such as Phaechamud et al. (2000) [51], who used a molar ratio of 1:1.2 for chitosan and citric acid, respectively, to prepare chitosan citrate dispersions. Similarly, Tanigawa (2008) [20] employed a 2% citric acid solution for a 1% chitosan dispersion, and Jiang (2023) [52] utilized approximately 1.9 mol of citric acid per 1 mol of glucosamine unit, also at 50 °C. Our findings therefore, suggest that the need for a higher molar ratio in the case of citric acid is primarily related to its polyprotic nature, which provides multiple protons and a stronger buffering capacity, resulting in lower pH values and enhanced neutralization of the chitosan amino groups. This interpretation is consistent with the literature, supporting the conclusion that ionic interactions and acid–base equilibria between chitosan and citric acid are predominant under the experimental conditions [53]. In addition, Chen et al. (1994) [54] reported that differences in apparent viscosity among chitosan–acid systems at the same pH can be attributed not only to electrostatic effects but also to steric hindrance exerted by bulkier carboxylate anions. Thus, steric hindrances of polycarboxylate anions, such as citrate, may play a role, but they are considered secondary to the acid–base equilibrium. These interactions likely facilitate colloidal dispersion and solubilization behavior, particularly when higher molar ratios and elevated temperatures are employed. In a study, Amorim (2016) [45] investigated the dispersibility of chitosan with identical concentrations of citric acid and lactic acid. In contrast to lactate, citrate anions are more tightly held in the electrical double layer of chitosan colloidal particles, partially shielding the positive charges of chitosan. This results in a weakening of the intra-chain and inter-chain electrostatic repulsion, thus leading to a lower dispersibility of this biopolymer in aqueous media containing citric acid compared to lactic acid. As a result, it is shown that the dispersibility of chitosan is not only related to the acid concentration but also to the type of acid.

Table 2 shows the K_a_ values for the acids used in this work. The lowest pK_a_ value was observed in glutamic acid (pK_a1_ = 2.13, K_a1_ = 7.41 × 10^−3^). The second lowest pK_a_ value was observed in citric acid (pK_a1_ = 3.13, K_a1_ = 7.41 × 10^−4^). Lactic acid has the third lowest pK_a_ value with 3.86 (K_a1_ = 1.38 × 10^−4^). The highest pK_a_ value was observed in acetic acid with 4.76 (K_a_ = 1.74 × 10^−5^) [48]. Based on the pK_a_ values, similar behavior was anticipated from the acids used in this study. However, citric acid exhibited distinct behavior compared to the other acids. The reason for the differing molar ratio required for chitosan–citric acid gels lies in the tricarboxylic structure of citric acid. This polyprotic nature, rather than steric hindrance, is considered the predominant factor underlying the need for a higher acid ratio. Using a higher amount (1.2 mol) of citric acid ensures that sufficient molecules are available to overcome steric hindrance and achieve complete protonation. Additionally, elevated temperature was applied during gel preparation to accelerate the dissolution of chitosan and to facilitate interaction with citrate ions by overcoming kinetic limitations that are prominent at room temperature. While steric hindrance of bulkier carboxylate anions may contribute to these observations, the dominant mechanism remains the polyprotic acid–base equilibrium.

### 2.2. Characterization of Chitosan Salts

#### 2.2.1. ^1^H Nuclear Magnetic Resonance (NMR) Analysis

**Figure 2:**
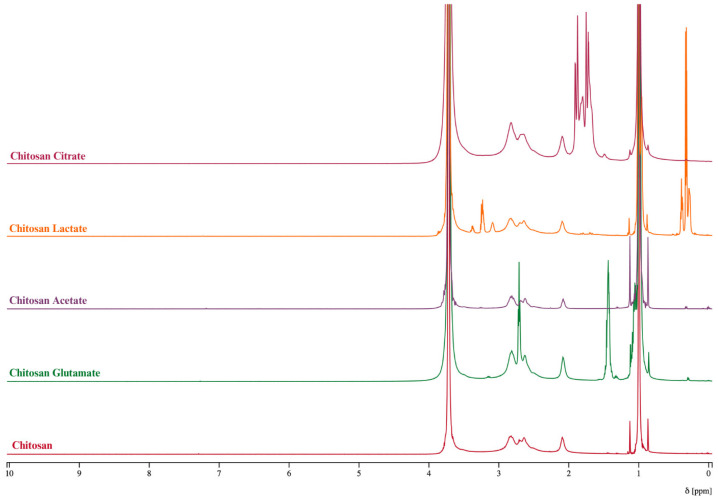
^1^H NMR spectra of chitosan and chitosan salts.

Preparation of the dispersions was performed using D_2_O. The proton NMR spectra of chitosan and its salts (chitosan lactate, chitosan acetate, chitosan citrate, and chitosan glutamate) are shown in Figure 2. The ^1^H NMR spectra of chitosan and its salts are essential for evaluating structural characteristics, particularly when analyzing modifications resulting from salt formation. All spectra exhibit a characteristic group of overlapping multiplets between δ 3.0 and 4.0 ppm, corresponding to the H-2 through H-6 protons of the glucosamine backbone. These signals are present across all samples, reflecting the conserved polysaccharide structure of chitosan and its typical spectral behavior in aqueous solution [55]. Unmodified chitosan lacked any distinct aliphatic signals below δ 2.5 ppm. In contrast, supporting that these downfield resonances in the salts result from the counter-ions associated through ionic interactions rather than covalently introduced functional groups. The observed chemical shifts, multiplicities, and coupling constants are in line with earlier reports and validate the structural identities of the chitosan salts. In the chitosan–acetate spectrum, a sharp singlet at δ 2.00 ppm is observed, corresponding to the methyl group of the acetyl moiety, confirming the presence of the acetate salt [55]. For chitosan-lactate, a well-resolved doublet at δ 1.30 ppm (J = 2.8 Hz) is attributed to the methyl group (CH_3_) of the lactic acid anion. These features are consistent with known NMR signatures of lactate ions associated with polysaccharides through protonation and ionic interactions, rather than covalent ester grafting. Similarly, the chitosan citrate spectrum produces distinct peaks in the 2.59–2.72 ppm range, which can be assigned to the methylene groups of the citrate anion [56]. For chitosan glutamate, signals corresponding to the α-CH proton of the glutamate appear around 3.75–3.85 ppm, while the β- and γ-CH_2_ protons resonate within the 2.00–2.40 ppm range [57]. These chemical shifts are highly specific, and they can provide reliable confirmation of salt formation and structural integrity rather than chemical modification [58].

#### 2.2.2. FTIR Analysis

**Figure 3:**
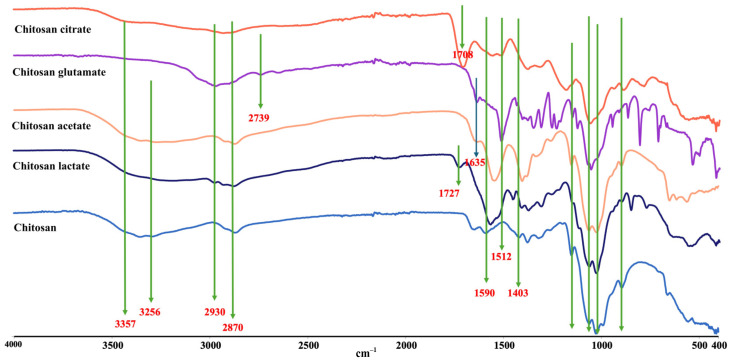
FTIR spectra of chitosan and chitosan salts.

The Fourier-transform infrared (FTIR) spectra of pure chitosan and its organic acid salts, recorded in the region of 4000–400 cm^−1^, revealed clear structural modifications resulting from protonation, salt formation, and possible crosslinking processes (Figure 3). Pure chitosan displayed a broad band around 3350 cm^−1^ due to the O–H and N–H stretching vibrations, as well as inter-hydrogen bonding between chitosan molecules [59,60,61]. In contrast, the amide-related N–H stretching vibration, which typically appeared broad in pure chitosan, shifted to around 2930 cm^−1^ in all salt spectra. This shift indicated the protonation of the amino groups (-NH_2_ → -NH_3_^+^) as a result of their interaction with organic acids. The characteristic peaks of pure chitosan, particularly the –CH_2_OH stretching vibration at approximately 1400 cm^−1^, disappeared in the salt spectra. This absence signified structural alterations due to salt formation [58,62].

In the glutamic acid salt, a distinct peak appeared at 1512 cm^−1^, attributed to the amine group of glutamic acid, confirming its incorporation into the polymeric structure. Similarly, the citrate salt exhibited a C=O stretching vibration at 1708 cm^−1^, while the lactate salt showed a similar peak at 1727 cm^−1^. These peaks highlighted the persistence of carboxylic acid groups within the structure, supporting the conclusion that partially free carboxylic acid groups remained unreacted [60]. In addition to these findings, the downward shift in the amino bending vibration from 1590 cm^−1^ in pure chitosan suggested the formation of -NH_3_^+^ groups in all chitosan salts. This shift was particularly important as it directly supports protonation and the formation of ionic bonds. Furthermore, the reduced intensity of carboxylic acid peaks in salt spectra indicated their involvement in interactions with chitosan chains [60,63].

The observed spectral changes confirmed that the organic acid salts of chitosan were formed through the protonation of amino groups and interactions with the carboxylic functionalities of the acids. These interactions resulted in structural modifications, including ionic bonding and potential covalent interactions, which significantly influence the physical and chemical properties of chitosan. Such changes are critical as they can modify key properties like dispersibility, mechanical strength, and water absorption capacity, thereby enhancing the material’s functionality.

#### 2.2.3. Thermogravimetric Analysis

**Figure 4:**
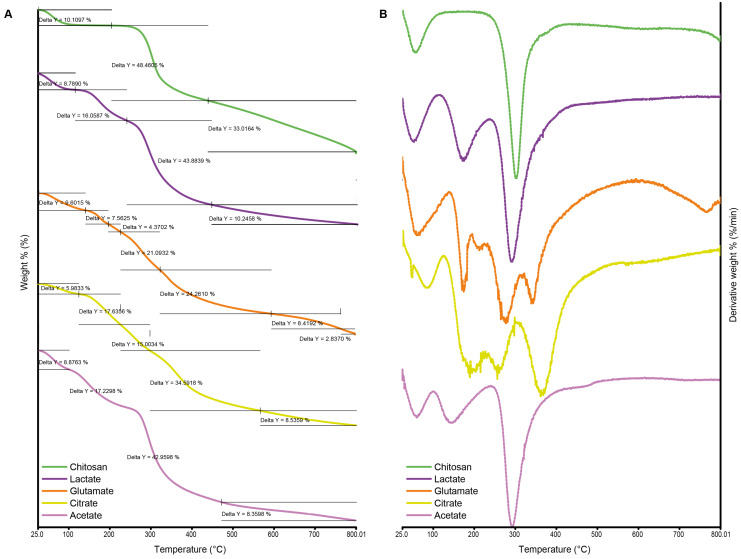
Thermogravimetric analysis (TGA) (**A**) and derivative thermogravimetry (DTG) (**B**) thermograms of chitosan and chitosan salts.

The weight loss rate of pure chitosan and the chitosan salts determined by TGA and DTG thermograms are given in Figure 4A and Figure 4B, respectively. The thermal decomposition behavior of chitosan and its derivatives played a crucial role in understanding their thermal stability and degradation mechanisms. In this study, the TGA data of acetate, glutamate, lactate, citrate salts, and pure chitosan were comparatively evaluated. The observed weight loss in chitosan and its derivatives indicated a multi-step degradation mechanism influenced by increasing temperature. For all samples, the initial weight loss, typically between 5 and 10%, occurred at low temperatures (100–150 °C). This loss was primarily associated with the removal of physically adsorbed water on the material’s surface, with weight loss values recorded as 9.6% for glutamate, 8.78% for lactate, 5.98% for citrate, and 10.1% for pure chitosan [63,64,65].

At moderate temperatures (200–400 °C), a second weight loss stage was observed, corresponding to the thermal decomposition of organic components and the removal of low-stability functional groups. The acetate salt exhibited a weight loss of 17.23% in this range, whereas the glutamate salt showed a comparatively lower loss of 7.56%. This difference suggested that the glutamate salt had a more stable structure, capable of withstanding higher temperatures before degradation. The main decomposition phase occurred between 300 and 500 °C, where the most significant weight loss was recorded due to the breakdown of the polymer backbone. At this stage, the lactate salt showed a weight loss of 43.88% and acetate 42.96%, which were close to each other, while glutamate exhibited a more controlled two-step degradation of 21.09% and 24.26%, respectively. These findings highlighted that lactate and acetate salts experienced more pronounced polymer chain degradation, whereas glutamate demonstrated a more gradual and complex decomposition profile. At high temperatures exceeding 600 °C, the removal of carbonized residues became evident. Pure chitosan exhibited a primary weight loss of 48.46%, followed by a residual carbon content of 33.01% [65]. This behavior can be attributed to the larger molecular structure and lower thermal stability of pure chitosan compared to its derivatives. On the other hand, citrate salt displayed a multi-step degradation pattern, leaving behind a relatively lower residue of 8.53%, indicating a more thermally stable behavior in the final stages. The varying decomposition behaviors of chitosan salts reflected differences in their thermal stability and chemical composition. Glutamate and lactate salts demonstrated better thermal stability at higher temperatures, making them more suitable for applications requiring temperature resistance. In contrast, acetate and citrate salts exhibited multi-step weight loss patterns, indicating more complex degradation mechanisms across different temperature ranges.

#### 2.2.4. XRD Analysis and Crystallinity Index (CrI) Determination

**Figure 5:**
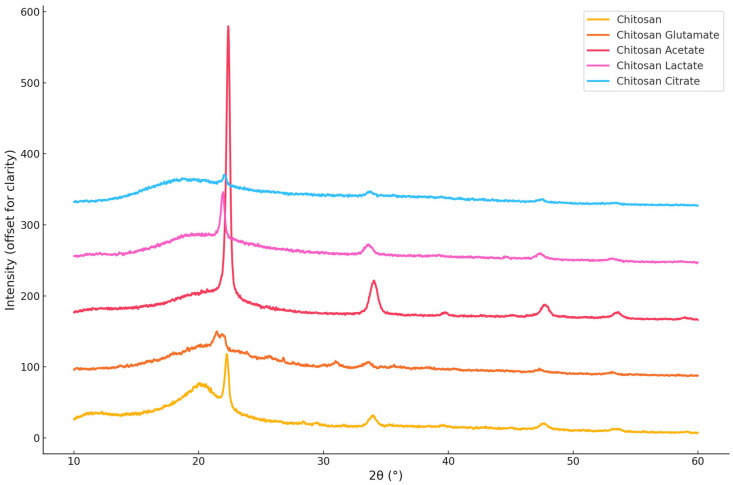
X-ray diffraction (XRD) patterns of chitosan and chitosan salts.

The crystalline structure of chitosan and its organic acid salts was evaluated using XRD, and the CrI was calculated with the Match! software (Version 4.0, Crystal Impact, Bonn, Germany). XRD graph of the salts is illustrated in Figure 5. Pure chitosan exhibited a broad diffraction peak around 2θ ≈ 20°, with a CrI value of 28.85%, confirming its characteristic semi-crystalline nature [58]. Chitosan acetate showed the highest CrI value (36.68%), indicating that acetate ions promote a relatively ordered molecular structure through stabilizing interactions with the chitosan backbone. This result is consistent with the findings of Nunthanid (2004) [33], who reported that acetate salts tend to retain crystallinity. In contrast, chitosan glutamate (27.74%) and chitosan lactate (25.63%) exhibited broader and less intense diffraction peaks, reflecting a more amorphous nature. These results align with earlier studies [16,62], which suggested that salt formation with glutamic and lactic acids disrupts hydrogen bonding and decreases structural order. Chitosan citrate showed a CrI value of 29.61%, slightly higher than pure chitosan. However, its more diffuse diffraction pattern suggests that, while the multivalent nature of citric acid may contribute to some degree of local ordering via ionic crosslinking, the overall structure remains largely amorphous [18,66]. These findings suggest that the type of organic acid used does not significantly affect the crystal organization of chitosan but leads to structurally distinct salt forms in varying degrees of order.

### 2.3. pH Determination

**Figure 6:**
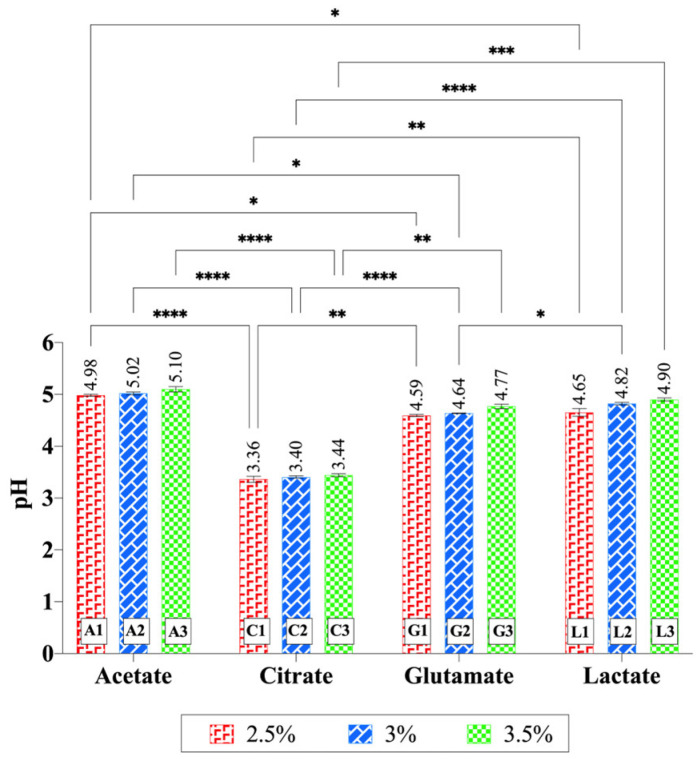
pH values of chitosan gels. Increasing chitosan concentration slightly elevated the pH values, although this change was not statistically significant within each acid type. Significant differences were observed between acid types at the same chitosan concentration. In particular, citrate-based hydrogels exhibited significantly lower pH values compared to the other acid types across all concentration levels. Statistical analysis was performed using two-way ANOVA followed by Tukey’s multiple-comparisons test (* *p* < 0.05, ** *p* < 0.01, *** *p* < 0.001, **** *p* < 0.0001).

As expected, the pH values of all gels were acidic, ranging from 3.36 to 5.10 (Figure 6). The pH of the gels increased with higher chitosan concentrations, though the change was not statistically significant (*p* > 0.05). Significant differences were also observed between acid types at the same chitosan concentrations. In particular, gels containing citrate exhibited lower pH values compared to the other acid types across all concentration levels (at least *p* < 0.01). Moreover, in the 2.5% chitosan hydrogels, significant differences were found between acetate–glutamate (*p* < 0.05) and acetate–lactate (*p* < 0.05). In the 3% chitosan hydrogels, a significant difference was detected between acetate–glutamate (*p* < 0.05) and glutamate–lactate (*p* < 0.05). The pK_a_ values of the acids used—acetic acid (4.76), citric acid (3.13, 4.76, 6.40), glutamic acid (2.13, 4.31, 9.67), and lactic acid (3.86)—reflect their varying acidic strengths and ionization behaviors. However, interpreting gel pH values solely based on pK_a1_ is an oversimplification, since polyprotic equilibria, the acid load per gram of gel, and ionic strength also play important roles. Acetic acid, a weak monoprotic acid, exhibits moderate acidity due to the electron-donating effect of its methyl group. Citric acid, a polyprotic acid, has its first proton (pK_a1_ = 3.13) as the most acidic, influenced by the electron-withdrawing effects of neighboring functional groups, and its additional dissociation steps contribute to a strong buffering capacity. For glutamic acid, the α-carboxyl group (pK_a1_ = 2.13) is the most acidic, due to the electron-withdrawing influence of the adjacent amino group, while the side-chain carboxyl group (pK_a2_ = 4.31) is less acidic. Lactic acid (pK_a1_ = 3.86) shows moderate acidity, with its hydroxyl group stabilizing the conjugate base through electron withdrawal [67]. In this study, pH was measured by dispersing 1 g of gel in 30 mL of water, a procedure commonly reported in the literature. This dilution corresponds to ~5.6 × 10^−3^ M (for 3% chitosan hydrogels), at which secondary dissociation steps can be neglected. Importantly, direct pH measurements performed in the gels without dilution gave comparable values, supporting the reliability of this method for comparative purposes. Based on the first pK_a_ values, the lowest pH was expected to be observed in chitosan glutamate. However, as shown in Figure 6, the lowest pH was observed in chitosan citrate (*p* < 0.001). This result can be attributed to the polyprotic nature and free carboxylic acid groups of citric acid, as verified by FTIR findings, which provide additional protons to the gel. Thus, the lower pH of citrate gels reflects not only pK_a1_ but also the buffering effects of polyprotic acids and their counter-ion interactions.

In a related study, a citric acid-crosslinked PVA/nano-silver hydrogel was prepared, and it was reported that some carboxylic acid groups of citric acid did not link to the polymer during the reaction. These free carboxylic acid groups imparted pH sensitivity to the hydrogel [68]. This confirms the compatibility of our findings with the literature. The second lowest pH was observed for chitosan glutamate, with an average pH of 4.67, followed by chitosan lactate at 4.79. The highest average pH (5.04) was observed for chitosan acetate, as expected. These pH values align well with the pK_a_ values of the acids used in the experiments, further validating the observed trends.

### 2.4. Rheological Properties and Spreadability

**Figure 7:**
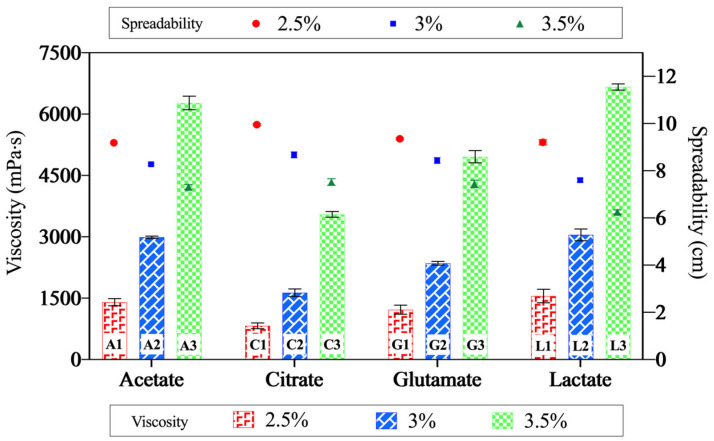
The relationship between viscosity and spreadability of the gels. The data points represent spreadability, and the bars indicate viscosity values. This dual representation highlights the inverse correlation between these two properties—gels with higher viscosity exhibit lower spreadability, while those with lower viscosity demonstrate greater spreadability.

The effect of acids on the flow characteristics of the chitosan gels was evaluated through viscosity and spreadability measurements, with the results demonstrated in Figure 7. Rheological studies using a rotational viscometer revealed that the chitosan acetate and the chitosan lactate gels exhibited similar and relatively high viscosities, whereas the citric acid-based gels demonstrated the lowest viscosity. Figure 7 illustrates the relationship between spreadability and viscosity. The spreadability values ranged from 6.25 to 9.95 cm, showing an inverse correlation with viscosity—higher viscosity formulations exhibited lower spreadability. Additionally, an increase in polymer concentration led to a decrease in gel spreadability [69].

The observed differences in viscosity among the chitosan gels, despite having the same chitosan concentration, can be attributed to factors such as the ionic strength of the acids, the functional groups in their structures, their hydrogen bonding capacities, and the degree of crystallinity of the resulting chitosan salts [41,54,70,71,72]. The ability of acids to interact with the amino groups of chitosan is also a significant factor in these variations [26]. Glutamic acid, with its two carboxylic acid groups and one amino group, exhibits high ionic strength [73,74]. Citric acid, containing three carboxylic acid groups capable of undergoing multiple protonation reactions, significantly increases the solution’s ionic strength and has the highest ionic strength among the acids tested [75]. Conversely, acetic acid and lactic acid, each with a single carboxylic acid group, exhibit weaker ionic strength as they provide only one proton to the medium [54,75]. In accordance with our findings, Soares et al. (2021) [76] showed that the rheological characteristics (consistency index and behavior index) of chitosan dispersions prepared with acetic acid, propionic acid, lactic acid, and glycolic acid, each having a single carboxylic acid group, were similar to each other.

Morariu et al. (2012) [41] linked the lower viscosity values observed at low chitosan concentrations to the weak polyelectrolyte nature of acetic acid. It was found that the electrostatic repulsion between the -NH_3_^+^ groups of glucosamine units was stronger in solvents with low ionic strength, leading to increased chain flexibility and, consequently, higher viscosity in chitosan dispersions. In acidic aqueous media with lower ionic strength, intermolecular interactions between chitosan chains promote the formation of aggregates, which further contribute to higher viscosity values. As the ionic strength increases, the pH of the solution decreases [77], which can cause breaks in the long polymer chains, resulting in lower viscosity values [78].

However, there were differences between the pH values of the gel formulations, which may have partially affected the viscosity. Nevertheless, although the gels prepared with acetic, lactic, and glutamic acids had similar pH values (Figure 6), they showed different viscosity values. This result suggests that the organic acid type had an effect independent of pH. Indeed, it was reported that viscosity values changed depending on the type of acid used in the chitosan dispersions prepared at the same pH, and explained this difference with steric and electrostatic effects of different acids [54]. Therefore, this study reveals that the rheological properties of chitosan gels are influenced not only by pH differences but also by the species of organic acid used.

The viscosity variations among chitosan gels are influenced by multiple factors, including crystallinity, acid type, and ionic strength. Chitosan acetate gels, despite having the highest CrI (36.68%), exhibit high viscosity due to strong hydrogen bonding between acetate ions and chitosan chains, enhancing network cohesion [79]. Conversely, chitosan citrate gels, with a CrI of 29.61%, show lower viscosity, likely because the high ionic strength of citric acid disrupts polymer chain interactions, reducing molecular weight and viscosity [79]. Interestingly, chitosan lactate gels, possessing the lowest CrI (25.63%), display high viscosity, attributed to free hydroxyl groups in lactic acid that enhance hydrogen bonding and promote a more entangled polymer network [17].

Tovar (2020) [40] investigated the rheological properties of chitosan (medium molecular weight, 90% degree of deacetylation) in lactic acid and acetic acid and reported that lactic acid yielded higher viscosity values. They attributed this to the ability of lactic acid and chitosan to form stronger hydrogen bonds and polar interactions, which enhance chain flexibility. Similarly, Romanazzi (2009) [80] prepared dispersions containing chitosan (1%) and aqueous solutions (1%) of various acids. Using the falling ball method, they found that the viscosities of chitosan lactate, chitosan acetate, and chitosan glutamate were 100, 40, and 23 mPa·s, respectively. These findings align with our results, confirming the consistency of our observations with the existing literature.

Rheograms are commonly used to determine whether a fluid exhibits Newtonian or non-Newtonian flow behavior [81]. The Power Law model (τ = K · γ^n^) was applied using the viscosity and shear stress (τ) data of the gels. The shear rate (γ) was calculated for each sample, and then ln(τ) and ln(γ) values were obtained by logarithmic transformation. According to these values, linear regression was applied, and flow behavior index (n), consistency coefficient (K), and R^2^ coefficient indicating the compatibility of the model were calculated for each gel (Table 3). The n value in all gels is below 1, which indicates that all of the samples exhibit pseudoplastic (shear-thinning) behavior. The model fit is quite high: R^2^ is calculated as >0.999 in all samples. The highest flow behavior index (n) was found to be 0.9675 for 2.5 citrate gel; this gel is closest to n ≈ 1 and shows less shear-thinning properties. The lowest n value was observed in L3 and calculated as 0.8017, which indicated a more pronounced shear-thinning behavior. Looking at the coefficients (K), the lowest K value was again calculated as 744.04 in C1 gel, while the highest K value was calculated as 4063.73 in L3. This showed that the L3 gel had a higher initial viscosity. These results have made it possible to evaluate the rheological behavior of gels not only qualitatively but also quantitatively. The high compatibility and stable parameters of the Power Law model reveal that this model was a suitable tool for the flow characterization of gels. These results confirm that the gels exhibit non-Newtonian flow characteristics. The observed decrease in viscosity with increasing shear rate is consistent with pseudoplastic flow behavior [82]. El-Hefian (2010) [64] demonstrated that a 1% chitosan–acetic acid dispersion (10 cP) exhibited non-Newtonian, shear-thinning (pseudoplastic) flow properties. Similarly, do Amaral Sobral (2022) [23] reported that chitosan dispersions (1–2.5%) prepared with aqueous acetic acid solutions also exhibited pseudoplastic flow behavior. Our findings align well with the previous studies, further validating the pseudoplastic nature of the chitosan gels.

### 2.5. Short-Term Physical Stability

**Figure 8:**
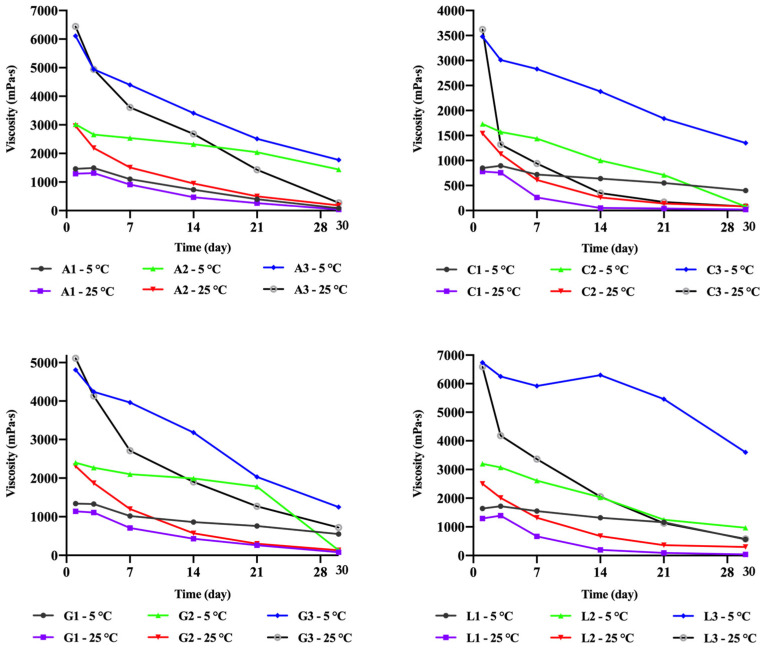
Change in the viscosity of the chitosan gels depending on time.

The short-term physical stability of chitosan gels was assessed by monitoring changes in viscosity over 30 days (Figure 8). The results revealed that gels stored at room temperature experienced a faster decline in viscosity, indicating that temperature directly affects polymer degradation. Low-concentration chitosan gels stored at room temperature nearly reverted to a liquid state by the end of the 30-day period. In contrast, gels stored at 5 °C exhibited slower viscosity changes, with the exception of chitosan acetate gels. Interestingly, the lactate gel containing 3.5% chitosan (L3 at 5 °C) showed only a slight decrease in viscosity within 14 days, suggesting that chitosan lactate was relatively more stable than the other gels. Preliminary studies also indicated that viscosity increased over time in gels containing 5% chitosan. This observation suggests that protonation by acids continues in high-concentration gels, leading to an increase in viscosity. Conversely, in low-concentration systems, where dispersibility is complete, viscosity decreases over time. Additionally, the chitosan citrate gels tended to degrade more rapidly than the others. This could be attributed to the higher number of carboxylic acid groups in its structure. These groups may interact with the hydroxyl groups of chitosan, breaking glycosidic bonds and accelerating chitosan degradation [29].

While previous studies have attributed the decrease in the viscosity of chitosan dispersions during storage to polymer degradation, some researchers have explained this trend as being caused by changes in macromolecular chain conformations and the degree of structuring. However, the latter phenomenon cannot account for the nearly complete dilution observed in concentrated polymer dispersions during long-term storage. Over time, it has been suggested that the decrease in the viscosity of chitosan dispersions in acetic acid is caused by an excess of acid in the system. While this may not pose a problem for short-term storage, over the long term, it leads to significant dilution and degradation of the polymer structure, resulting in a decrease in the molar mass of chitosan [83].

Furuike et al. investigated the effect of acid concentration on gel stability and pH. In their study, a chitosan hydrogel prepared with acetic acid was neutralized with sodium hydroxide. Subsequently, this hydrogel and pure chitosan powder were tested for dispersibility in acidic media with pH values of 3 and 5. While the chitosan hydrogel dispersed in both media, pure chitosan powder did not disperse at pH 5. A decrease in viscosity of approximately 20% was observed in the chitosan hydrogel within 2 days, after which it remained stable for 5–6 days. In contrast, a 60% decrease in viscosity was noted in the chitosan acetate dispersion within 7 days. It was concluded that using excess acid accelerates this degradation process. Consequently, it was recommended to prepare a chitosan hydrogel first and subsequently convert it into a dispersion to achieve long-term physical stability. Furthermore, the longer stability of high-concentration chitosan gels was likely related to their higher pH values [35]. Conversely, the rapid loss of physical stability in low-concentration gels was attributed to their lower pH values (Figure 6). In particular, the low pH values of citric acid–based gels may have contributed to their faster deterioration tendency. Nevertheless, the fact that other acid salts with similar pH values exhibited different stability profiles indicates the independent contribution of the organic acid type.

### 2.6. Cytotoxicity Studies

**Figure 9:**
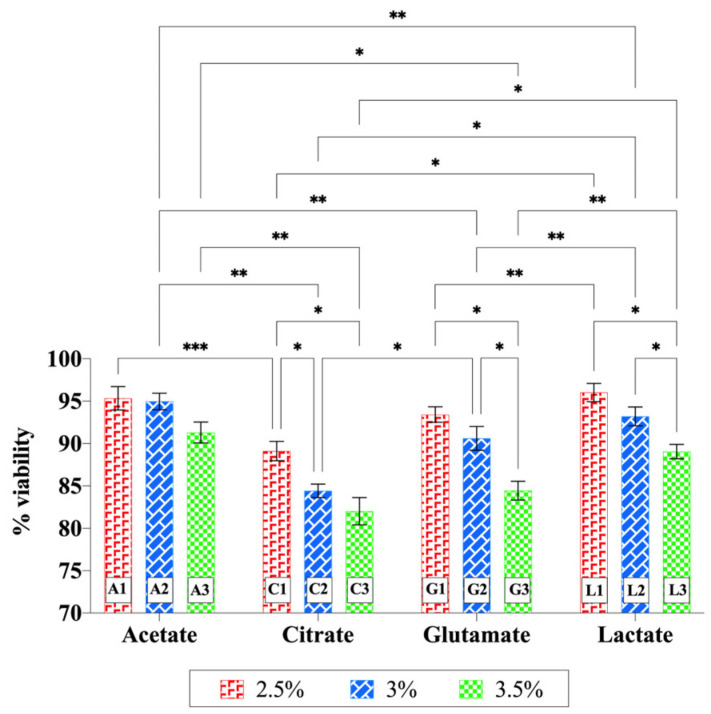
Cytotoxicity of chitosan hydrogels prepared with different acid types on HaCaT cells. Statistical analysis was performed using two-way ANOVA followed by Tukey’s multiple-comparisons test (* *p* < 0.05, ** *p* < 0.01, *** *p* < 0.001).

The cytotoxicity of chitosan hydrogels was evaluated using the HaCaT cell line. The cell viability percentages of the formulations did not fall below 70%, even at the highest concentration tested (1000 µg/mL) (Figure 9). Therefore, lower concentrations were not tested, and due to the lack of cytotoxic effects, IC_50_ (half-maximal inhibitory concentration) values could not be determined. Two-way ANOVA showed that both acid type (*p* = 0.0010) and chitosan concentration (*p* = 0.0024) had significant main effects on cell viability, while the interaction between the two factors was not significant (*p* = 0.2264). When each acid type was evaluated individually, increasing chitosan concentration in the acetate hydrogels did not result in a significant change in viability. In contrast, significant decreases in cell viability were observed at higher concentrations of the citrate, glutamate, and lactate hydrogels (*p* < 0.05). When acid types were compared at the same chitosan concentration, hydrogels containing citrate and glutamate in particular yielded lower viability values in many cases, indicating that acid type also significantly influenced cell viability. Overall, higher chitosan concentrations (especially 3.5%) and specific acid types (citric acid and glutamic acid) exerted a greater decreasing effect on cell viability. The citrate-based hydrogels exhibited significantly lower pH values (Figure 7) and correspondingly lower cell viability compared to the other hydrogels. This suggests that the observed cytotoxicity may be partially related to the low pH. However, lower viability was also observed in the glutamate-containing hydrogels, suggesting that additional acid-dependent mechanisms (e.g., ionic interactions or cellular stress responses) may also be involved. Despite these differences, all chitosan hydrogels prepared with different acid types maintained cell viability above 80%, indicating their overall biocompatibility.

Previous studies have also supported the high biocompatibility and low cytotoxicity of chitosan-based systems. Luo et al. (2022) [1] showed that chitosan hydrogels prepared with acetic acid exhibited no toxicity in fibroblast cells and demonstrated high biocompatibility. Sun et al. (2023) [84] reported that chitosan hydrogels cross-linked with citric acid displayed low cytotoxicity and good biocompatibility. Cevher et al. (2015) [31] tested both chitosan glutamate and chitosan chloride salts in the Calu-3 cell line and found that both forms maintained approximately 80% cell viability up to 1 mg/mL. In another study, Pieklarz et al. (2021) [85] demonstrated that chitosan lactate-based hydrogels were highly biocompatible in HT-29 cells and even promoted cell proliferation, achieving over 100% viability (112.1% and 101.8%). In contrast, cell viability was lower in systems containing chitosan hydrochloride (91.0% and 98.2%), which was attributed to the lower pH of hydrochloric acid compared to lactic acid.

In conclusion, the chitosan-based hydrogels evaluated in this study did not exhibit significant cytotoxic effects on HaCaT cells, with cell viability remaining above 80% in all groups. Nevertheless, in addition to increasing chitosan concentration, the type of acid used and the resulting pH differences appear to play a role in the variations in cell viability that were observed.

### 2.7. Swelling Studies

**Figure 10:**
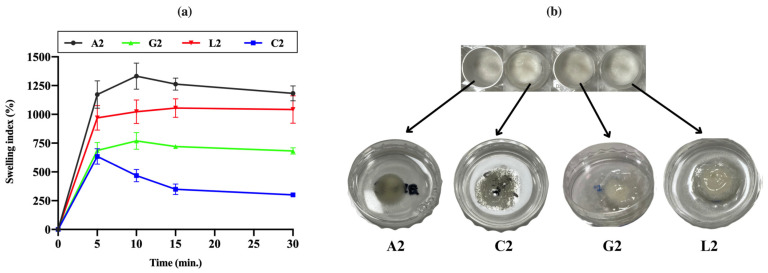
(**a**) Swelling profiles of the dried chitosan gels. (**b**) Freeze-dried gels and their state after 15 min in the swelling medium. The dried gels swelled rapidly by taking on water. While chitosan citrate gel loses its physical integrity relatively faster than others, chitosan lactate was the most stable.

The swelling index of lyophilized chitosan gels was measured based on weight changes. Chitosan citrate was observed to crumble easily after lyophilization, with edges breaking apart even during the weighing process. The experiments continued until the freeze-dried gels lost their structural integrity or completely transformed into a gel. The swelling profiles of the dried gels are shown in Figure 10a. As the lyophilized products absorbed water from the medium, some began to disintegrate or gel from the edges. All products reached their maximum swelling level within 15 min (Figure 10b). However, chitosan citrate started to disperse rapidly, leading to weight reductions after the 5th minute. It was concluded that gels prepared with citric acid exhibited a lower swelling index due to their faster disintegration and dispersibility. A positive correlation was generally observed between the swelling properties of the gels and their viscosity [86]. These findings suggest that chitosan citrate has a weaker physical structure compared to other chitosan salts. Similarly, previous studies have shown that chitosan gel formulations with lower viscosity swell and degrade more quickly, which also results in a faster release profile when used as drug carriers [26]. Swelling behaviors may also be partially affected by the pH values of the gels. However, different swelling profiles were observed even in acetate, lactate, and glutamate gels, which had similar pH values. This supports the view that the counter-ion type shapes swelling behaviors independently of pH.

Huanbutta et al. prepared spray-dried salts of chitosan (45 kDa and 200 kDa; 87–89% DD) using aspartic acid, glycolic acid, glutamic acid, and lactic acid. These chitosan salts were formulated into tablets, and their swelling properties were evaluated. The swelling percentages were ranked as follows: glycolate > lactate > acetate > glutamate. The hydroxyl groups in glycolate and lactic acid facilitated water absorption, resulting in greater swelling. In contrast, acetate and glutamate, containing amine (-NH_2_) and carboxyl (-COOH) groups, promoted intermolecular interactions or attractive forces between polymer chains, leading to lower swelling. The erosion profiles of the tablets further supported these swelling results [87]. Qu (2020) [72] reported that chitosan glycolate and chitosan lactate hydrogels exhibited similar water absorption capacities at pH 7.4, which was attributed to the similar chemical structures of the acids, differing only by a methyl group. In another study, crosslinked chitosan–terephthaldehyde showed weaker swelling properties compared to chitosan acetate films. This was associated with reduced chain flexibility caused by an increased number of crosslinking nodes in the polymer network, resulting in decreased water penetration and a less hydrophilic character [88]. Our findings align with these reports, demonstrating consistency with the existing literature.

### 2.8. Bioadhesion Studies

**Figure 11:**
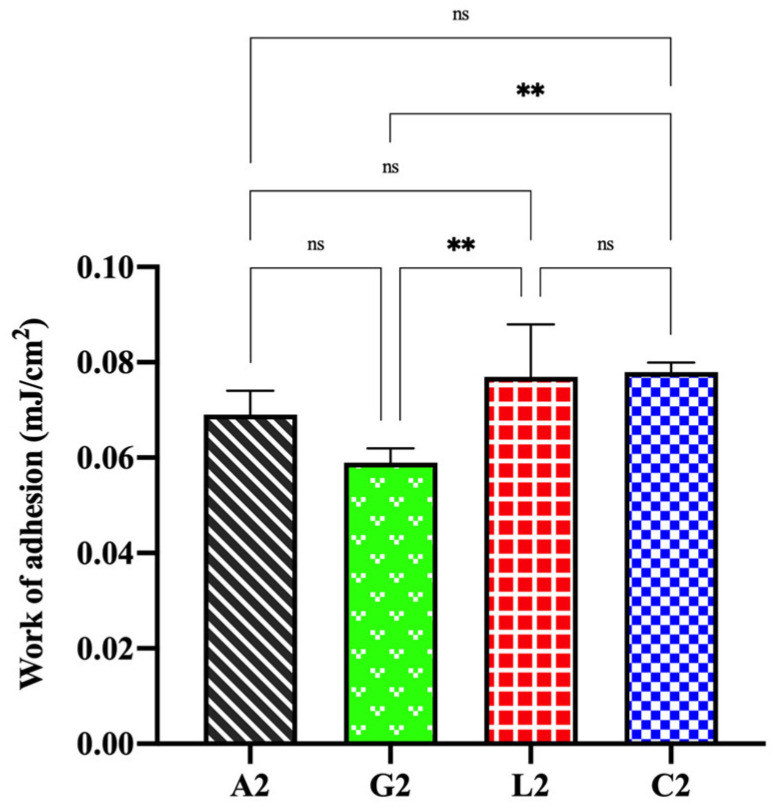
Bioadhesive properties of chitosan salt gels (ns: not significant, ** *p* < 0.01).

Bioadhesion refers to the adhesive interaction between a biological substrate and a formulation. The adhesive ability of a formulation applied to the skin or mucosa is critical for its effectiveness, as it enhances penetration, absorption, and bioavailability by allowing the formulation to remain at the target area for a longer time [58,89,90]. Chitosan, derived by partial deacetylation of chitin, is a widely used polycationic polymer with excellent bioadhesive properties, making it valuable in biomedical, pharmaceutical, biotechnological, and cosmetic applications [91,92]. The bioadhesive properties of chitosan arise from ionic interactions, hydrogen bonding, and physical entanglement enabled by its linear, flexible structure. Additionally, factors such as MW, DD, and chemical modifications significantly influence its adhesive characteristics [58,93]. In this work, the bioadhesive ability of four organic acid salts of chitosan was evaluated. Bioadhesion studies using Balb/c mouse skin revealed that chitosan glutamate gel generally exhibited lower bioadhesive properties compared to the others (*p* < 0.05). Gels with high swelling and viscosity, such as chitosan lactate, demonstrated superior bioadhesiveness. Interestingly, despite its low swelling index and viscosity, chitosan citrate also displayed strong bioadhesive properties. While a linear relationship between water absorption and adhesion is generally expected [94], the low swelling index of the chitosan citrate was attributed to its poor physical stability in aqueous media. As the dried chitosan citrate rapidly absorbed water, it began dispersing and degrading within 5 min, resulting in lower swelling percentages. However, in the semi-solid gel state used during the bioadhesion study, no disintegration or structural degradation occurred. The enhanced bioadhesive property of chitosan citrate, despite its weak swelling index and viscosity, may be attributed to the high hydrogen bonding capacity provided by its carboxyl groups. Consequently, the work of adhesion values were determined as follows: C. citrate ≥ C. lactate > C. acetate > C. glutamate (Figure 11).

It was reported that there is generally a linear relationship between the viscosity of gels and their adhesiveness [95]. However, in our study, only chitosan citrate demonstrated high bioadhesive values despite its low viscosity. This could be attributed to the increased chain flexibility in linear polymers, which enhances bioadhesion. Additionally, the molecular size may influence bioadhesive ability. Larger molecular structures facilitate greater interpenetration and physical entanglement, leading to improved bioadhesive properties [96,97]. The relatively larger molecular structure of chitosan citrate compared to other salts may explain its superior bioadhesion. In a previous study, the swelling and bioadhesive properties of buccal tablets containing chitosan glutamate and chitosan chloride were investigated. The findings showed that chitosan glutamate exhibited lower swelling but higher bioadhesive ability [58]. Cerchiara et al. demonstrated that chitosan lactate exhibited stronger bioadhesive properties than chitosan glutamate. However, the mucoadhesive ability of chitosan glutamate significantly improved when interacting with mucin dispersions. Furthermore, bioadhesion was generally higher at physiological pH than at pH 5.5 [26]. These findings indicate that, beyond the physicochemical properties of chitosan, factors such as the route of administration and environmental conditions play crucial roles in determining its adhesive ability.

## 3. Conclusions

This study suggests that the choice of organic acid plays an important role in influencing the physicochemical properties, stability, and bioadhesion of chitosan hydrogels. While lactate gels appeared to provide the most balanced performance under the tested conditions characterized by high viscosity, stability, and bioadhesion, citrate gels tended to show lower stability and lower cell viability but comparatively higher adhesive strength. Glutamate gels presented intermediate features, while acetate gels combined relatively high viscosity with moderate swelling and adhesion. Although acid type and pH influenced cell viability to some extent, all hydrogels exhibited high biocompatibility, with cell viability remaining above 80%. These findings indicate that acid selection provides a practical tool to tailor the functionality of chitosan-based gels beyond polymer concentration or crystallinity alone. It should be noted that the ionic strength was not kept constant across all formulations, since the aim of this study was to compare the intrinsic effect of different organic acids under stoichiometric conditions. In addition, it should be noted that the properties of the gels are also affected by pH. However, different behaviors were observed even in gels with similar pH values, indicating that the counter-ion type provides an independent contribution. Future studies should differentiate these two effects through systematic comparisons under equal pH conditions. Nevertheless, variations in ionic strength may also contribute to the observed differences in gel properties, and this aspect should be systematically addressed in future investigations. Moreover, it will be important to assess the impact of different chitosan molecular weights and degrees of deacetylation, as these parameters may further influence the properties and performance of chitosan salt-based gels. In addition, the present study was limited to physicochemical, rheological, and short-term stability evaluations performed under controlled in vitro conditions. The results therefore provide preliminary guidance for the rational design of chitosan-based gels as bioadhesive systems. Future work should extend the scope to include long-term stability (particularly for gels prepared with higher chitosan concentrations or higher molecular weight chitosan), drug release kinetics, and in vivo assessments in order to confirm their translational potential. In addition, the present findings may guide the design of bioadhesive formulations, including mucosal drug delivery platforms, where tailoring gel properties by acid selection could enhance therapeutic performance.

## 4. Materials and Methods

### 4.1. Materials

Chitosan (DD ≥ 90%, L-MW, 10–20 kDa; provided by the manufacturer) was purchased from BBI Chemicals (Shanghai, China). Phosphate-buffered saline tablets (pH 7.4), glutamic acid, lactic acid (88–92%), ascorbic acid, glacial acetic acid, and 3-(4,5-dimethylthiazol-2-yl)-2,5-diphenyltetrazolium bromide (MTT) were purchased from Sigma (St. Louis, MO, USA). Citric acid (anhydrous) was purchased from Balmumcu Kimya (Istanbul, Türkiye). Cell culture reagents were obtained from Gibco (Paisley, UK). All chemicals used in this project were analytical grade. Ultrapure water (resistivity 18.2 MΩ·cm, obtained from an Elga Flex 3 system, Veolia, High Wycombe, UK) was used during all the studies.

### 4.2. Preparation of Chitosan Hydrogels

Chitosan-based gels were prepared using various organic acids, including acetic acid, glutamic acid, ascorbic acid, and lactic acid, at a 1:1 molar ratio of chitosan to organic acid [26,28,43]. For citric acid, a 1.2 molar ratio was used [51]. The organic acids were precisely weighed based on their molar mass and dissolved in distilled water. Chitosan was then added to the solution and stirred at room temperature using a mechanical stirrer (PZR2020, Heidolph Instruments, Schwabach, Germany) until a clear gel was obtained. In contrast, chitosan citrate gels were homogenized at 50 °C. In summary, all components (chitosan, organic acid, and water) were weighed on a weight basis. Organic acid amounts were calculated according to the molar ratio of the chitosan monomer unit (179.17 g·mol^−1^) to each acid (1:1 for acetic, lactic, glutamic, and ascorbic acids; 1:1.2 for citric acid). After weighing chitosan and the respective acid, distilled water was weighed to reach a total of 100 g formulation. Their pH values were measured as part of the physicochemical characterization (see Section 4.4). The resulting gels were protected from light and stored at 4 °C in a refrigerator for subsequent analyses. Gels prepared with ascorbic acid were excluded from the study due to poor physical stability, as they transitioned from gel to sol phase within a few days. The composition of the chitosan-based gels is summarized in Table 1.

### 4.3. Characterization of Chitosan Salts

In order to characterize the chitosan salts, 1 g from each gel (3% of chitosan gels; A2, C2, G2, and L2) was individually weighed in a round plastic mold (Ø: 17 mm) and lyophilized (Toros Freeze Dryer, TRS 2-2V, Teknosem, Istanbul, Türkiye) at −60 °C for 48 h. The obtained freeze-dried gels were carefully removed from the mold. Then, the dried gels were crushed using a blade mill (Sinbo, Istanbul, Türkiye). FTIR spectra between 400 and 4000 cm^−1^ with 4 cm^−1^ resolution (Spectrum two with ATR equipment, PerkinElmer, Waltham, MA, USA) and proton nuclear magnetic resonance ^1^H NMR spectra using D_2_O for the solutions (Bruker Avance NEO 500 MHz NMR, Bruker Corporation, Billerica, MA, USA) were taken for the pure chitosan and chitosan salts. Thermogravimetric analyses were performed using ~4 mg of sample, with a ramp of 10 °C per minute under 20 mL/min Nitrogen gas between 25 °C and 800 °C (TGA 8000, PerkinElmer, Waltham, MA, USA). XRD patterns of the chitosan salts were obtained from a powder X-ray diffractometer (Bruker D2 Phaser, Karlsruhe, Germany) between 0 and 90° 2θ values with Cu Kɑ radiation (λ = 1.5406 nm).

### 4.4. pH Determination

The pH of the gel formulations was measured using a calibrated pH meter (HI 83141, Hanna Instruments, Woonsocket, RI, USA) at room temperature. For this purpose, 1 g of each gel was weighed into a beaker and stirred in 30 mL of distilled water until fully dispersed [98]. The pH meter probe was immersed in the dispersion and left in place until the reading stabilized.

### 4.5. Spreadability

To assess the spreadability of the gels, one gram of gel was placed at the exact center of a glass plate (20 × 20 cm^2^). Another glass plate of the same dimensions was carefully placed on top. After 1 min, the diameter of the spread gel was measured at multiple points [69]. The studies were performed at room temperature.

### 4.6. Rheological Investigations

The studies were conducted using a rotational viscometer (Brookfield DV2T-RV, Middleboro, MA, USA) at room temperature, with minor modifications to previous methods [99]. Initially, the viscosities of the gels were measured at a constant speed of 25 rpm. Subsequently, the changes in viscosity and shear stress as a function of increasing shear rate were evaluated. The SC27 spindle was used for all measurements, which were performed in triplicate. Furthermore, the Power Law model was applied using the obtained data to evaluate the flow characteristics [23].

### 4.7. Short-Term Physical Stability

The gel formulations were transferred into light-proof, air-tight containers and stored at 5 ± 3 °C and 25 ± 2 °C to assess their short-term physical stability. At predetermined time points (days 1, 3, 7, 14, 21, and 30), the stability of the gels was evaluated by measuring their viscosity using the rotational viscometer at room temperature. Additionally, the physical appearance of the gels was monitored visually.

### 4.8. Cytotoxicity Studies

Human–human keratinocyte cells (HaCaT) were cultured in high-glucose Dulbecco’s Modified Eagle Medium (DMEM, high glucose) supplemented with 10% (*v*/*v*) heat-inactivated fetal bovine serum, 2 mM L-glutamine, and a standard antibiotic–antimycotic mixture (penicillin 100 U/mL, streptomycin 100 μg/mL, amphotericin B 0.25 μg/mL). Cells were maintained at 37 °C in a humidified atmosphere containing 5% CO_2_ and were subcultured every three days to preserve exponential growth [100]. Cell viability was assessed using the MTT colorimetric assay. Briefly, HaCaT cells were seeded into 96-well plates at a density of 1 × 10^5^ cells/well and allowed to adhere for 24 h. The cells were then exposed to the test formulations. After the incubation period, the medium was replaced with 30 µL of MTT solution (0.5 mg/mL in sterile phosphate-buffered saline), and the plates were further incubated for 4 h at 37 °C. Formazan crystals were solubilized using 150 µL of isopropanol, and absorbance was measured at 570 nm with a microplate reader (SpectraMax i3, Molecular Devices, San Jose, CA, USA). Each experimental condition was tested in triplicate and repeated in at least three independent experiments.

### 4.9. Swelling Behavior

To investigate the swelling behavior of the gels (3% chitosan), the gels were lyophilized using the freeze dryer as previously described. The obtained freeze-dried gels were carefully removed from the mold and weighed (W_1_), allowing for the determination of water loss during the lyophilization process. The dried gels were then immersed in 5 mL of phosphate-buffered saline (PBS) in a Petri dish. At predetermined intervals, the PBS was carefully removed using a plastic pipette, and any surface moisture on the gels was gently blotted with filter paper before reweighing (W_2_). This process was repeated until no further weight increase was observed or the gel began to degrade or disperse. The swelling index of the gels was calculated using Equation (1) [101]. Each experiment was performed individually in triplicate at room temperature.

Swelling index (%) = [(W_2_ − W_1_)/W_1_] × 100. (1)

### 4.10. Ex Vivo Bioadhesion Study

The ex vivo adhesion investigation was performed in compliance with the National Institutes of Health (NIH) guidelines and the regulations outlined by the Istanbul Medipol University Ethical Council (approval number: E-38828770-772.02-6921). Shaved skin samples from Balb/c mice were used as biological substrates to determine the bioadhesive properties of the gels using a texture analyzer (TA.XTplusC, Stable Micro Systems, Haslemere, Surrey, UK). Prior to analysis, the skin samples were removed from the freezer, allowed to equilibrate at room temperature for approximately 30 min, and then securely wrapped around the probe (SNSP/10, Ø: 10 mm) of the device with a rubber band. One gram of gel (3% chitosan) was placed in a beaker, which was fixed on a heating plate (Mr-Hei Standard, Heidolph Instruments, Schwabach, Germany) using double-sided tape to prevent movement. Bioadhesion data were obtained by bringing the skin into contact with the gel for a specific duration and then separating them. The operating parameters were as follows: pretest speed—0.5 mm/s; test speed—0.1 mm/s; post-test speed—0.1 mm/s; applied force—0.5 N; and contact time—120 s [102]. The studies, conducted at 32 ± 2 °C to simulate biological conditions, were performed independently five times. The bioadhesion strength and work of adhesion were calculated using the device’s software (Texture Exponent 8.0.7.0) based on the corresponding equations.

Work of adhesion (mJ·cm^−2^) = AUC_1-2_/πr^2^ (2)

The “AUC_1-2_” and “r” indicated the area under the curve of the force-distance diagram and the radius of the device probe around which the mice skin was wrapped, respectively.

### 4.11. Statistical Analysis

All obtained data are presented as mean and standard deviation. For pH and cytotoxicity analyses, statistical significance was assessed by ordinary two-way ANOVA (factors: acid type and chitosan concentration), followed by Tukey’s multiple-comparisons test. For bioadhesion studies, the statistical significance of the findings was evaluated with one-way analysis of variance (ANOVA) followed by Tukey’s multiple-comparisons test. All analyses were performed using GraphPad Prism (version 9.0.1, La Jolla, CA, USA), and *p* < 0.05 was accepted as statistically significant.

## Figures and Tables

**Table 1:** Composition of gel formulations.

Material	A1	A2	A3	C1	C2	C3	G1	G2	G3	L1	L2	L3
Chitosan (%)	2.5	3	3.5	2.5	3	3.5	2.5	3	3.5	2.5	3	3.5
Acetic acid *	1: 1	1:1	1:1									
Citric acid *				1:1.2	1:1.2	1:1.2						
Glutamic acid *							1:1	1:1	1:1			
Lactic acid *										1:1	1:1	1:1
Distilled water (g) (qs)	100	100	100	100	100	100	100	100	100	100	100	100

* molar ratio of chitosan:acid. qs: quantum sufficient. All components (chitosan, organic acid, and water) were weighed on a weight basis. Organic acid amounts were calculated according to the molar ratio of the chitosan monomer unit (179.17 g·mol^−1^) to each acid.

**Table 2:** pK_a_ values of organic acid used [48].

Acid	pK_a1_	pK_a2_	pK_a3_	Strength (Acidic Protons)
Acetic Acid	4.76			Weak
Lactic Acid	3.86			Moderate
Glutamic Acid	2.13	4.31	9.67	Strongest for K_a1_, weakest for K_a3_
Citric Acid	3.13	4.76	6.40	Stronger for K_a1_, weaker for others

**Table 3:** Power Law parameters of gel samples and model compliance.

Formulation	Behavior Index (n)	Consistency Coefficient (K) (mPa·s^n^)	R^2^
A1	0.9003	1062.54	0.9996
A2	0.8369	1972.77	0.9993
A3	0.8124	3929.39	0.9997
C1	0.9675	744.04	0.9997
C2	0.9189	1325.92	0.9997
C3	0.8764	2584.21	0.9994
G1	0.9048	972.54	0.9997
G2	0.8846	1754.30	0.9994
G3	0.8392	3289.02	0.9995
L1	0.8945	1116.81	0.9995
L2	0.8335	1860.15	0.9992
L3	0.8017	4063.73	0.9996

## Data Availability

The original contributions presented in this study are included in the article. Further inquiries can be directed to the corresponding author.

## Abbreviations

The following abbreviations are used in this manuscript:

ANOVA	One-way analysis of variance
CrI	Crystallinity index
DD	Deacetylation degree
DMEM, High	High-glucose Dulbecco’s Modified Eagle Medium
DTG	Derivative thermogravimetry
FTIR	Fourier-transform infrared
H-MW	High molecular weight
HaCaT	Human keratinocyte cells
IC_50_	Half-maximal inhibitory concentration
L-MW	Low molecular weight
M-MW	Medium molecular weight
mPa·s	Milli-Pascal second
mr	Molar ratio
MTT	3-(4,5-dimethylthiazol-2-yl)-2,5-diphenyltetrazolium bromide
NIH	National Institutes of Health
NMR	Nuclear magnetic resonance
PBS	Phosphate-buffered saline
PVA	Polyvinyl alcohol
qs	Quantum sufficient
TGA	Thermogravimetric analysis
XRD	X-ray diffraction
